# Effects of time of initiation of antiretroviral therapy in the treatment of patients with HIV/TB co-infection: A systemic review and meta-analysis

**DOI:** 10.1016/j.amsu.2020.05.004

**Published:** 2020-05-16

**Authors:** Legese Chelkeba, Ginenus Fekadu, Gurmu Tesfaye, Firehiwot Belayneh, Tsegaye Melaku, Zeleke Mekonnen

**Affiliations:** a School of Pharmacy, Institute of Health, Jimma University, Jimma, Ethiopia; b School of Pharmacy, Institute of Health Sciences, Wollega University, Nekemte, Ethiopia; c Department of Pharmacy, College of Medicine and Health Sciences, Ambo University, Ambo, Ethiopia; d Department of Pharmacy, College of Medicine and Health Sciences, Dilla University, Dilla, Ethiopia; e School of Medical Laboratory Sciences, Institute of Health, Jimma University, Jimma, Ethiopia

**Keywords:** Immune reconstitution syndrome, HIV/TB, Antiretroviral therapy, AIDS, Tuberculosis

## Abstract

This systemic review and meta-analysis aimed to investigate the burden of tuberculosis immune reconstitution syndrome (TB-IRIS) and associated mortality to highlight the importance of future direction in preventing and treatment of TB-IRIS. Randomized clinical trials (RCTs) that compared early antiretroviral therapy (ART) versus late ART were included. PubMed, EMBASE, Science Direct and Cochrane Central Register of Controlled Trials electronic databases were searched. This meta-analysis included 8 RCTs with a total of 4, 425 participants. The result of analysis showed that early initiation of ART was associated with increase in TB-IRIS (RR = 1.83; 95% CI: 1.24–2.70, *p* = 0.002; I^2^ = 74%, *p* = 0.0007) and TB-IRIS associated mortality (RR = 6.05; 95% CI: 1.06–34.59, *p* = 0.04; I^2^ = 0%, *p* = 0.78). Early ART was associated with overall mortality compared with late ART initiation. Grade 3 or 4 adverse events, achieving lower viral load and development of new AIDS-defining illness were not associated with the time of ART initiation. Early ART in HIV/TB co-infected patients resulted conclusive evidence of increased TB-IRIS incidence and TB-IRIS associated mortality. Hence, the finding calls for clinical judgment as to the benefits of initiating ART earlier against the risk of TB-IRIS and associated mortality.

## Highlights

• Tuberculosis (TB) remains the largest cause of death among people living with HIV/AIDS.
• TB-associated immune reconstitution inflammatory syndrome (IRIS) is common among TB patients on ART.
• Evidences showed that there is still delay in initiation of ART in resource limited countries.
• The optimal timing for the initiation of ART in relation to TB therapy remains controversial.
• Early ART initiation among HIV/TB co-infected patients resulted in increased TB-IRIS incidence and mortality.

## 1 Introduction

Tuberculosis (TB) remains one of the most common opportunistic infections among people living with HIV/AIDS, especially among those with profound immunosuppression [1]. A pooled meta-analysis by Zheng et al. [2] including 272, 466 participants in the world revealed that the prevalence of TB/HIV co-infection was 31.25% in Africa, 17.21% in Asia, 20.11% in European, 25. 06%, in Latin American and 14.84% in United States. World health organization (WHO) 2016 also reported that the risk of developing TB was more likely in people living with HIV compared to those without HIV infection (9.6 million new cases of TB, of which 1.2 million (12.5%) were among people living with HIV) [3].

Integrated therapy is crucial for HIV-infected patients with TB as TB-related mortality in HIV-infected patients is high during the first few months of TB treatment [4]. Guidelines recommend that all HIV-infected TB patients should be commenced on ART irrespective of their CD4 count which has the potential to reduce mortality. Antiretroviral therapy (ART) should be given within 8 weeks of initiation of anti-tuberculosis treatment in TB patients with a CD4 count of ≥50 cells/mm^3^less than 50 cells/mm^3^, but it should be started within 2 weeks after the onset of anti-tuberculosis treatment in patients with CD4 less than 50 cells/mm^3^. In HIV patients with TB meningitis however, immediate ART initiation was associated with more severe adverse events, and should be delayed until the 8-12 weeks of anti-tuberculosis treatment [5]. However, there is insufficient evidences to support or refute a survival benefit conferred by early versus late ART initiation [6]. Although guidelines promote early initiation of ART, reliable evidence showed that there is still delay in initiation of ART in resource limited countries [7].

Despite the suggestion of randomized controlled trials (RCTs) and meta-analyses to initiate ART after 2 weeks of TB treatment, the delicate balance between the risk of morbidity and mortality in advance HIV diseases with potential occurrence of additive toxicity, drug-drug interactions, pill burdens and TB-IRIS remains challenging to clinicians and patients [1]. TB-associated immune reconstitution inflammatory syndrome (TB-IRIS) is common in patients with TB started on ART [8]. The incidence of TB-IRIS reported so far ranges from 11% to 45% [9]. Moreover**,** available evidences reported that TB-IRIS is usually self-limiting and patients should continue their anti-tuberculosis and ART treatments [10]. Although the optimal supportive treatment for TB-IRIS is not well understood, a RCT undergoing to determine the optimal regimen for TB-IRS management is currently underway [11, 12]. Currently available meta-analyses reported the overall mortality and incidences of IRIS associated with early ART versus late ART TB patients [13]. However, none of them reported the magnitude of death due to TB-IRIS. Therefore, to provide an up-to-date summary of reliable evidences on TB-IRIS associated deaths and other endpoints, we conducted systemic review and meta-analysis of RCTs evaluating the effectiveness and safety of early ART versus late ART in TB patients.

## 2 Materials and methods

The meta-analysis reported in accordance with the Preferred Reporting Items for Systematic Reviews and Meta-Analyses (PRISMA) guideline [14]. The work has been reported in line with a critical appraisal tool for systematic reviews that include randomized or non-randomized studies of healthcare interventions [15].

### 2.1 Search strategy

Four investigators (LCH, GF, GT and TM) independently searched electronic databases in PubMed, EMBASE, Science Direct and the Cochrane clinical trials database from inception to August 2016 using the terms *(“HIV” OR “human immunodeficiency virus” OR “AIDS” or “acquired immune deficiency syndrome” AND “ART” OR “HAART” or “highly active antiretroviral therapy” OR “Antiretroviral” AND “mycobacterium tuberculosis” OR “tuberculosis” OR “anti-tuberculosis” AND “early treatment” OR “immediate treatment” AND “delayed treatment” OR “differed treatment” AND “inflammatory reconstitution syndrome” OR “IRIS” OR “mortality”).* A manual search for additional relevant studies using references from retrieved articles was also performed. Conference abstracts and unpublished studies were excluded. We restricted the searches to human studies with English language placed on the searches. We followed Patient, Intervention, Comparison and Outcome (PICO) to identify relevant studies:

• Population (P): Patients with HIV/AIDS
• Exposure (E): Early initiation of ART
• Comparison (C): Late initiation of ART
• Outcome (O): Incidence of TB-IRIS and associated mortality

### 2.2 Types of studies, participants, and interventions

The same authors (LCH, GF, GT and TM) independently assessed the inclusion criteria and disagreement was solved by discussion. We included the studies if they met the following criteria: the study design was an RCT trial which compared initiating ART at different times after starting to receive TB medications (early versus late groups), reports at least one of the primary or secondary endpoints. The population comprised adults and adolescents (≥13 years) with confirmed HIV infection and diagnosed to have active TB (pulmonary and extra-pulmonary). Studies were excluded if the population were pregnant women, pediatrics, if there were incomplete information and no clear outcome comparison between competitors.

### 2.3 Data abstraction and quality assessment

The four independent authors extracted data from all eligible studies on to a standardized data abstraction sheet. We extracted information on the name of first author, year of publication, study deign and setting, types of patients included, number of participants (early/late), interval between start of TB therapy and ART therapy, ART regimen, TB regimen, duration of TB treatment, duration of follow up, age, gender, primary and secondary outcomes. The same authors independently assessed the included trials for bias according to the Handbook for Systematic Reviews of Interventions [16] and disagreement also resolved by discussion. The following parameters were assessed: sequence generation, allocation concealment, masking (blinding) of participants, personnel and outcome assessors, incomplete outcome data and selective outcome reporting. Other sources of bias were risk of bias related to the specific study design used or trial stopped early due to some data-dependent process or an extreme baseline imbalance in patients selected according to this hand book.

### 2.4 Outcome measures

The primary outcomes were the incidences of TB-IRIS and associated deaths reported by each trails. Other secondary outcomes considered were overall mortality, new AIDS-defining illness, TB treatment failure, and TB recurrence rate, incidence of grade 3 or 4 adverse events and rate of HIV RNA suppression to <400 copies/ml. Death due to TB-IRIS was defined in the same way as original articles. We used the definition used by the original papers, which were based on criteria from the International Network for the study of HIV immune reconstitution inflammatory syndrome for classification of TB immune reconstitution inflammatory syndrome [17]. Therefore, the following strict criteria were applied to diagnose TB-IRIS:

• Initial improvement of TB-related symptoms and/or radio-graphic findings after adequate anti-tuberculosis treatment for a certain time.
• Paradoxical deterioration of TB-related symptoms and/or radiologic findings at the primary or at new locations during or after anti-tuberculosis treatment.
• Absence of conditions that reduce the efficacy of anti-tuberculosis drugs (e.g., poor compliance, drug malabsorption, drugs side effects).
• Exclusion of other possible causes of clinical deterioration.

### 2.5 Statistical analysis

We followed the Cochrane hand book of data analysis and reported dichotomous outcome measures to assess the summary effects of treatment by calculated risk ratio (RR) with 95% CI. A random-effects model was used because of anticipated heterogeneity. Statistical heterogeneity among trials was expressed as the p-value (Cochran's Q statistic), where a p < 0.05 and I [2] statistic >50% indicated significant heterogeneity. A predefined sub-group analyses were done based on:

⁃ Effects of ethnicity of the study population (Africans versus Asians) on TB-IRIS and death due to TB-IRIS.
⁃ Effects of type of antiretrovirals used (‘‘D’’ drugs versus none ‘‘D’’ drugs) on TB-IRIS and death due to TB-IRIS.
⁃ Effect of immune status (CD4 counts ≥ 50 cells/mm^3^or < 50 cells/mm^3^) on overall mortality.

The analyses was carried out using Rev Man 5.3 software (The Nordic Cochrane Center, Denmark) to create a forest plot and a summary finding tables. The D drugs were stavudine and didanosine and non D drugs were zidovudine and tenofovir.

### 2.6 Ethics approval and consent to participate

Not applicable. The study was registered at researchregistry.com with a unique reference number of “reviewregistry863”

## 3 Results

### 3.1 Literature searches and selection

**Fig. 1:**
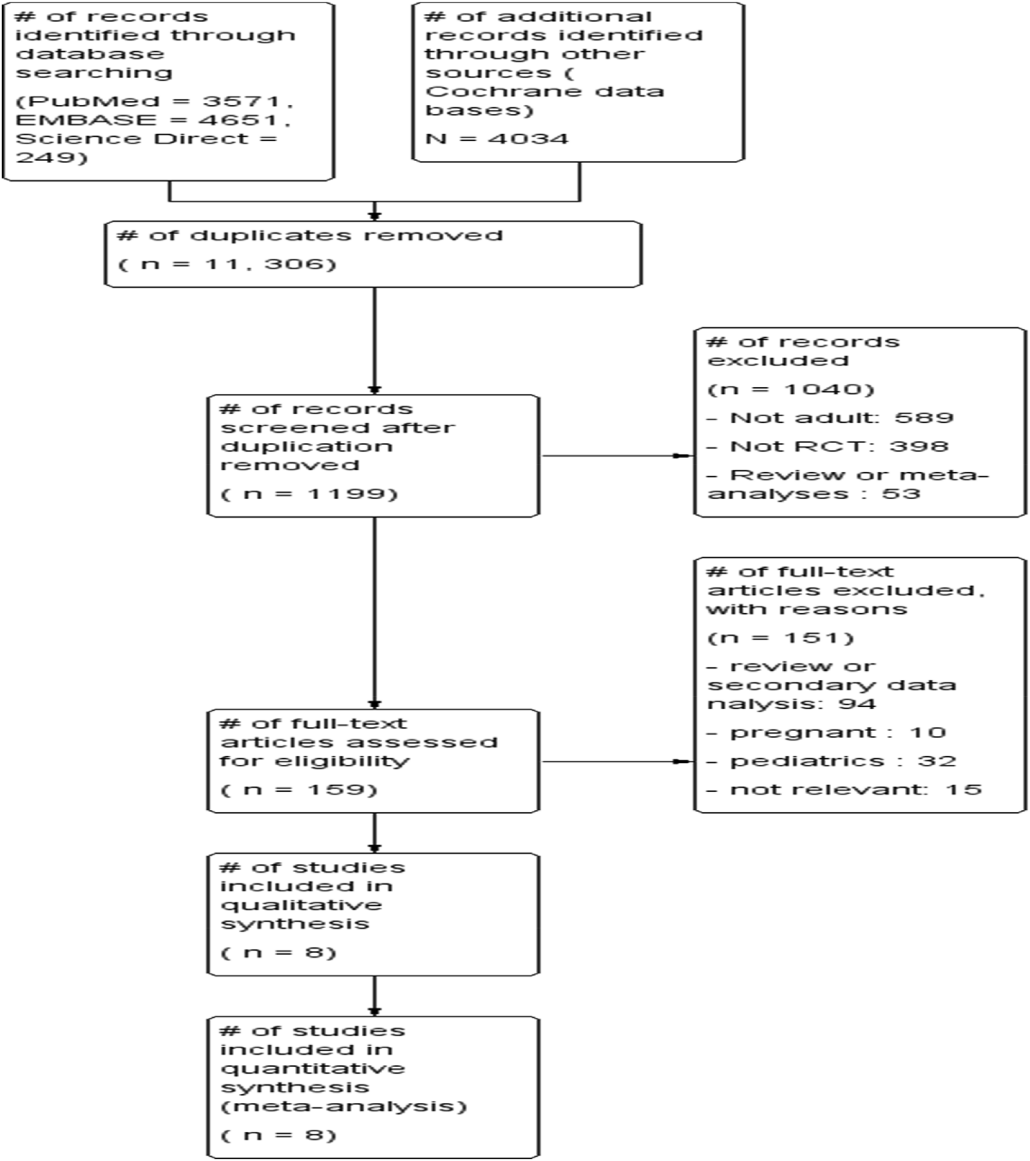
PRISMA flow diagram of included studies in the systematic review and meta-analysis of the effects of time of initiation of antiretroviral therapy in the treatment of adolescent and adult non-pregnant patients with HIV/TB co-infection.

Initial research of electronic databases such as PubMed, EMBASE, Science direct and Cochrane clinical trial databases yielded 12,505 articles, from which 1199 records remained after removing 11,306 duplications. Upon screening the articles, 1040 articles were further excluded; 598 were not adults, 389 articles were not RCTs, 53 articles were reviews or meta-analyses. Upon further access to the full texts of 159 articles, 151 articles were excluded for the following reasons; 94 review articles, 10 included pregnant women, 32 were about pediatric patients and 15 were not relevant. Finally, the remaining 8 full articles were included in both qualitative and quantitative analyses (Fig. 1).

### 3.2 Study characteristics

Eight RCTs published between 2009 and 2015 fulfilling the inclusion criteria were included in the final analyses [5, 8, [18], [19], [20], [21], [22], [23], [24], [25]]. The sample size of the included trials ranged from 70 [24] to 1675 [23] with a total number of 4425 patients, of which 2310 were assigned to the early ART group (1-4 weeks) and 2, 115 to late ART group (8-24 weeks). The trials were conducted in Sub-Saharan Africa, Asia, South America and United States between 2005 and 2014 and published between 2009 and 2015. The median/mean age of the patients included in the study ranges from 32 to 38 years. Patients in all included trails were treated for TB within a median duration of 6 months of standard short course anti-tuberculosis treatment; 2 months of isoniazid, rifampicin, ethambutol and pyrazinamide followed by 4 months of isoniazid, and rifampicin. All of the patients initiated with ART regimen of efavirenz with 2 backbones of nucleoside analogues with the exception of patients in one trial [24] in which patients received three nucleoside analogues (Table 1).

**Table 1:** Baseline characteristics of studies included in systemic review and meta-analysis of adolescent and adult non-pregnant patients with HIV/TB co-infection.

First Authors/year	Study design and settings	Types of patients	#participant (Early/late)	ART regimen	TB regimen	Duration of TB treatment	Early versus late start of ART after TB treatment	Duration of follow up	Median/median age(year)	Woman, %
Abdool, 2011 [SAPiT] [18]	RCT, open-label, TB clinics of south Africa	New patients, ambulatory patients of CD4+<500mm3	214/215	DDI + 3 TC + EFV	2RHZE/4RH 2RHZES/100days of continuation	6 months	4 weeks vs. 12 weeks	18 months	34.4	51.3
Amogne, 2015 [19]	RCT, open-label(multicenter), Hospital and clinics of Addis Abeba, Ethiopia	New patients, ambulatory patients of CD4+<200mm3	323/155	EFV+ 3 TC, and study site physician selection of ZDV,TDF and D4T	2RHZE/4RH	6 months	1–4 weeks vs. 8 weeks	12 months	35.3	49.7
Blanc, 2011 [CAMELIA] [20]	RCT, open-label (multicenter) five hospitals in Cambodia	New patients, inpatients and outpatients HIV and TB with CD4+<200mm3	332/329	D4T+3 TC + EFV.	2RHZE/4RH	6 months	2 weeks vs 8 weeks	25 months	35	35.7
Manosuthi, 2012 [TIME] [22]	RCT, open-label, Thailand	HIV/TB-co infected patients	79/77	TDF+3 TC + EFV	2RHZE/4-7RH	6–9 months	4 weeks vs.12 weeks	96 weeks	38	22.4
Shao, 2009 [24]	RCT, double-blinded Tanzania	-HIV-1 and tuberculosis (TB)-confected patients with no previous history of ART	35/35	ABC+3 TC + AZT	–	6 months	2 weeks vs.8 weeks	104 weeks	36	41
Sinha, 2012 [25]	RCT, open- label Delhi, India.	ART naïve HIV positive patients with active TB	88/62	D4T or AZT (ZDV) +3 TC + EFV	2 RHZE/4 RH	6 months	2–4 weeks vs.8-12 weeks	12 months	34.8	16
Mfinanga, 2014 [TB-HAART][23]	RCT, double-blinded international multi-centered study South Africa, Uganda Zambia Tanzania	Participants had to be at least 18 years of age, HIV positive, smear-positive and culture-positive for tuberculosis, with CD4 counts of 220 cells per μL or more, and no previous tuberculosis treatment in the preceding 2 year	834/841	AZT + 3 TC + EFV	2RHZE/4RH	6 months	2 weeks vs 6 months	24 months	32	40
Harvlir, 2011 [STRIDE] [21]	RCT, multi country, open label South Africa South America United States Asia	Patients 13 y of age or older with HIV-1 infection with aCD4+ T-cell count of0.250 × 109 cells/L who had not previously received ART and had confirmed or probable TB	405/401	EFV + TDF/FTC	2HRZE/4HR	6months	2 weeks vs. 8-12 weeks	25 months	34	38

Abbreviations: RCT, randomized clinical trial; TB, tuberculosis; HIV, human immunodeficiency virus; ART, antiretroviral therapy; 3 TC, lamivudine; AZT(ZDV), Zidovudine; TDF, tenofovir; D4T, stavudine; DDI, didanosine; EFV, efavirenz; ABC, abacavir; R, Rifampin; H, isoniazid; Z, pyrazinamide; E, ethambutol; S streptomycin.

### 3.3 Risk of bias assessment

**Fig. 2:**
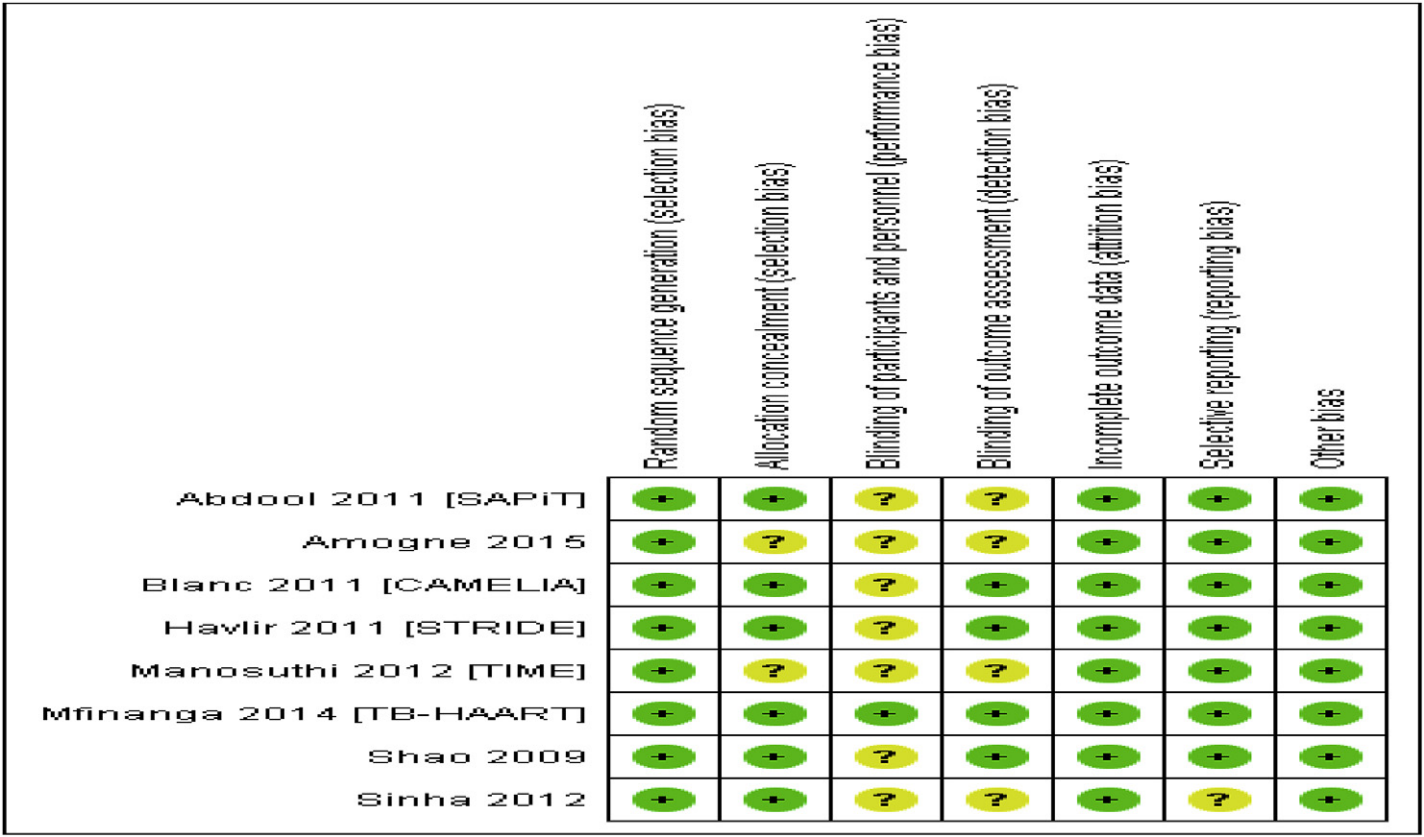
The risks of bias assessment of the studies in the systematic review and meta-analysis of the effects of time of initiation of antiretroviral therapy in the treatment of adolescent and adult non-pregnant patients with HIV/TB co-infection.

Allocation sequence generation was adequate in all trials and allocation concealment was adequate in 6 trials and unclear in the remaining 2 [19, 22]. Blinding of participants and personnel was unclear in 7 of the trials and adequate in the remaining one [5, 23], whereas blinding of outcomes assessment was adequate in 4 trials and unclear in the remaining 4 trials [18, 19, 22, 25]. The rate of discontinuation of therapy was significantly higher in early ART treatment group compared with late treatment group in one study [25], (*p = 0.007*) which subjected the potential for attrition bias. No evidence of selective reporting and other bias observed according to the judgment of the authors (Fig. 2).

### 3.4 Primary outcomes

#### 3.4.1 Death due to TB-IRIS

**Fig. 3:**
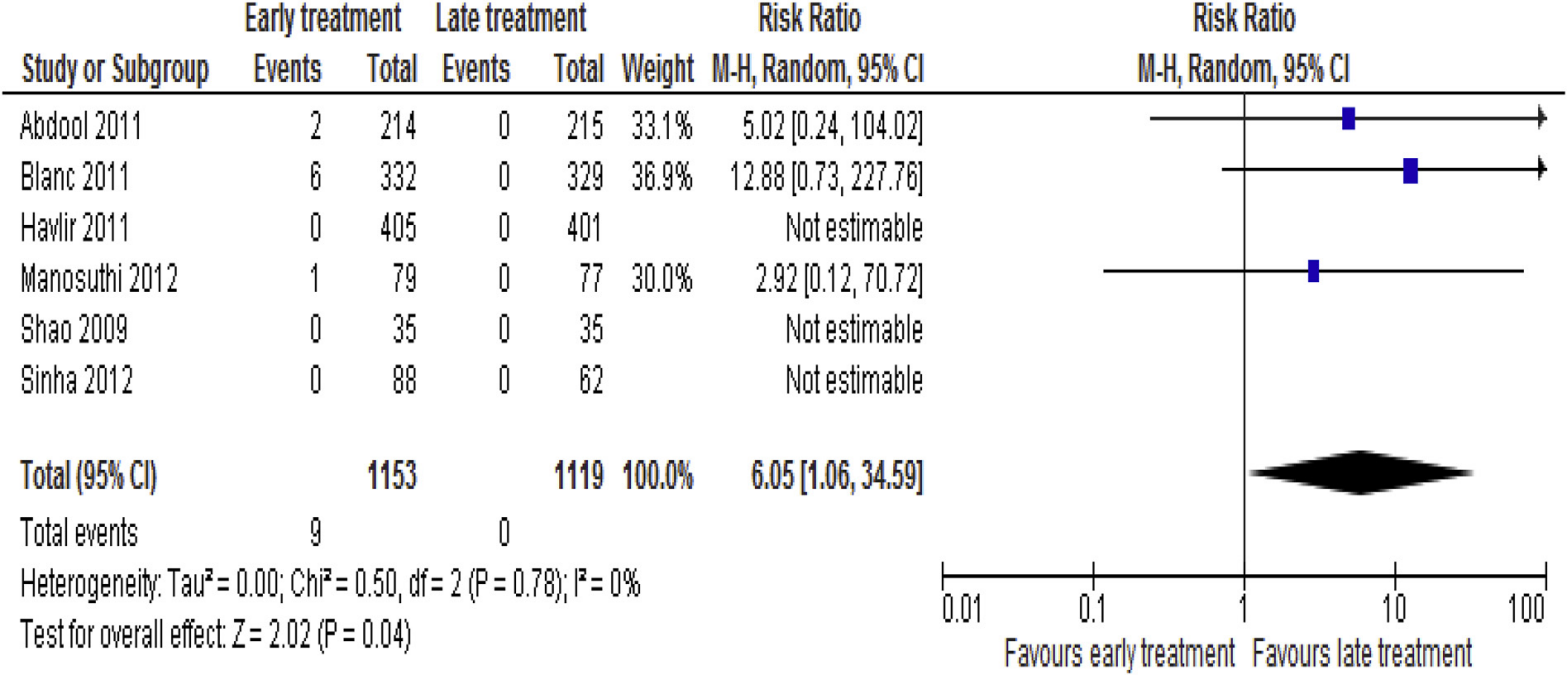
Death due to TB-IRIS associated with the effects of time of initiation of antiretroviral therapy in the treatment of adolescent and adult non-pregnant patients with HIV/TB co-infection.

Data were available for six [8, 18, [20], [21], [22], 24, 25] trials on death due to TB-IRIS including 2, 272 patients. Nine (0.78%) patients died due to TB-IRIS in the early ART group compared to zero in the late ART group (RR = 6.05; 95% CI: 1.06–34.59, p = 0.04; I^2^ = 0%, p = 0.78) (Fig. 3).

The absolute risk reduction was 0.78%, which means that for 143 patients treated with ART 8-24 weeks after TB treatment in patients co-infected with HIV and TB, one case of IRIS related mortality would be prevented. Subgroup analysis showed that early ART was associated with a higher incidence of death due to TB-IRIS than late ART for Asian patients (RR = 6.63; 95% CI: 0.78–55.93, p = 0.08). However, no significant difference observed in African patients between early ART and late ART (RR = 5.02; 95% CI: 0.24–104.02, p = 0.30). Similarly, early ART was associated with higher rate of death due to TB-IRIS than late ART for patients received ‘D’ drugs (Stavudine, D4T or Didanosine, DDI) (RR = 8.25; 95% CI: 1.03–66.32, p = 0.05), but no significant difference observed between early ART and late ART for patients received non ‘D’ drugs (TDF or AZT) (RR = 2.92; 95% CI: 0.12–70.72, p = 0.51).

#### 3.4.2 Incidence of TB-IRIS

**Fig. 4:**
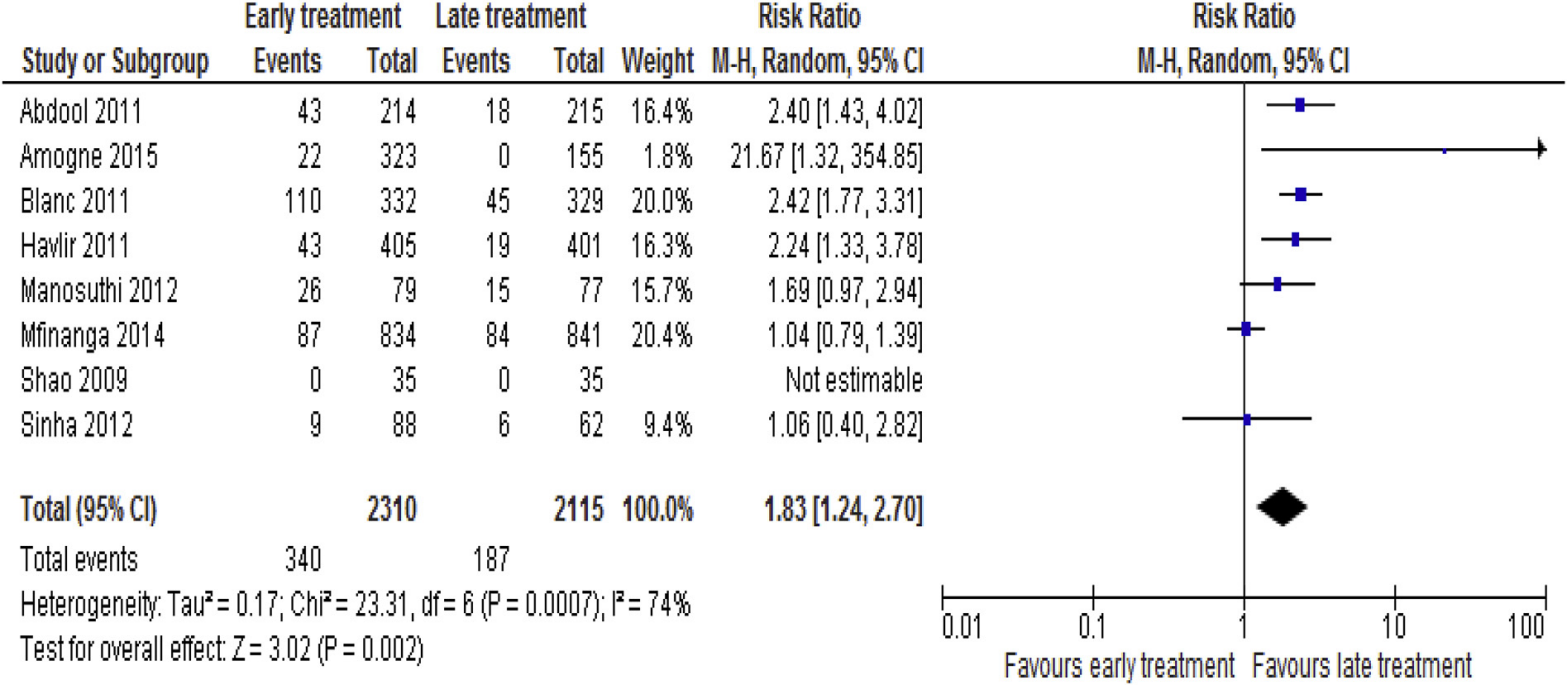
Incidences of TB-IRIS associated with the effects of time of initiation of antiretroviral therapy in the treatment of adolescent and adult non-pregnant patients with HIV/TB co-infection.

TB-IRIS data were available for 8 [8, [18], [19], [20], [21], [22], [23], [24], [25]] trials included 4425 participants. Among 2310 patients received early ART, 430 (14.7%) developed TB-IRIS compared with 187 (8.8%) in the late ART group (RR = 1.83; 95% CI: 1.24–2.70, p = 0.002; I^2^ = 74%, p = 0.0007) (Fig. 4).

The absolute risk reduction was 5.9%, which means that for every 100 patients initiated with ART late in the course of TB treatment (after 8-24 weeks of TB treatment), about 6 TB-IRIS cases would be averted. In another word, 17 patients needed to be initiated with ART late in the course of TB treatment to prevent one case of TB-IRIS. Up on subgroup analysis, we found that early ART was associated with higher incidence of TB-IRIS than late ART in Asians (RR = 1.93; 95% CI: 1.29–2.87, p = 0.001). Similarly, early ART was associated with higher rate of TB-IRIS than late ART for patients given ‘‘D″ drugs (RR = 2.42; 95% CI: 1.85 = 3.16, p < 0.00001).

### 3.5 Secondary outcomes

#### 3.5.1 Overall mortality

**Fig. 5:**
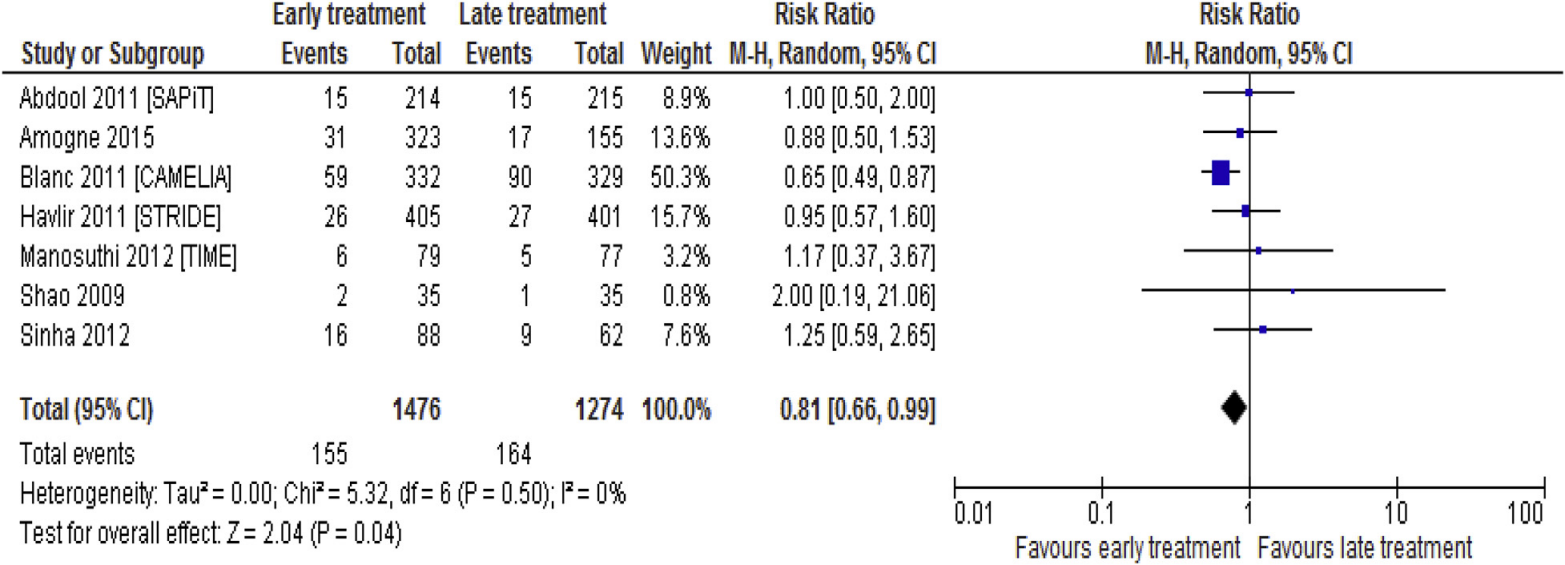
Overall mortality associated with the effects of time of initiation of antiretroviral therapy in the treatment of adolescent and adult non-pregnant patients with HIV/TB co-infection.

Data on overall mortality was available for 7 [5, [18], [19], [20], [21], [22], 24, 25] trails including 2750 participants. Early ART initiation was associated with lower overall mortality rate compared to late ART initiation (RR = 0.81; 95% CI: 0.66–0.99, p = 0.04; I^2^ = 0%, p = 0.50) among HIV-TB co-infected adult patients (Fig. 5).

**Fig. 6:**
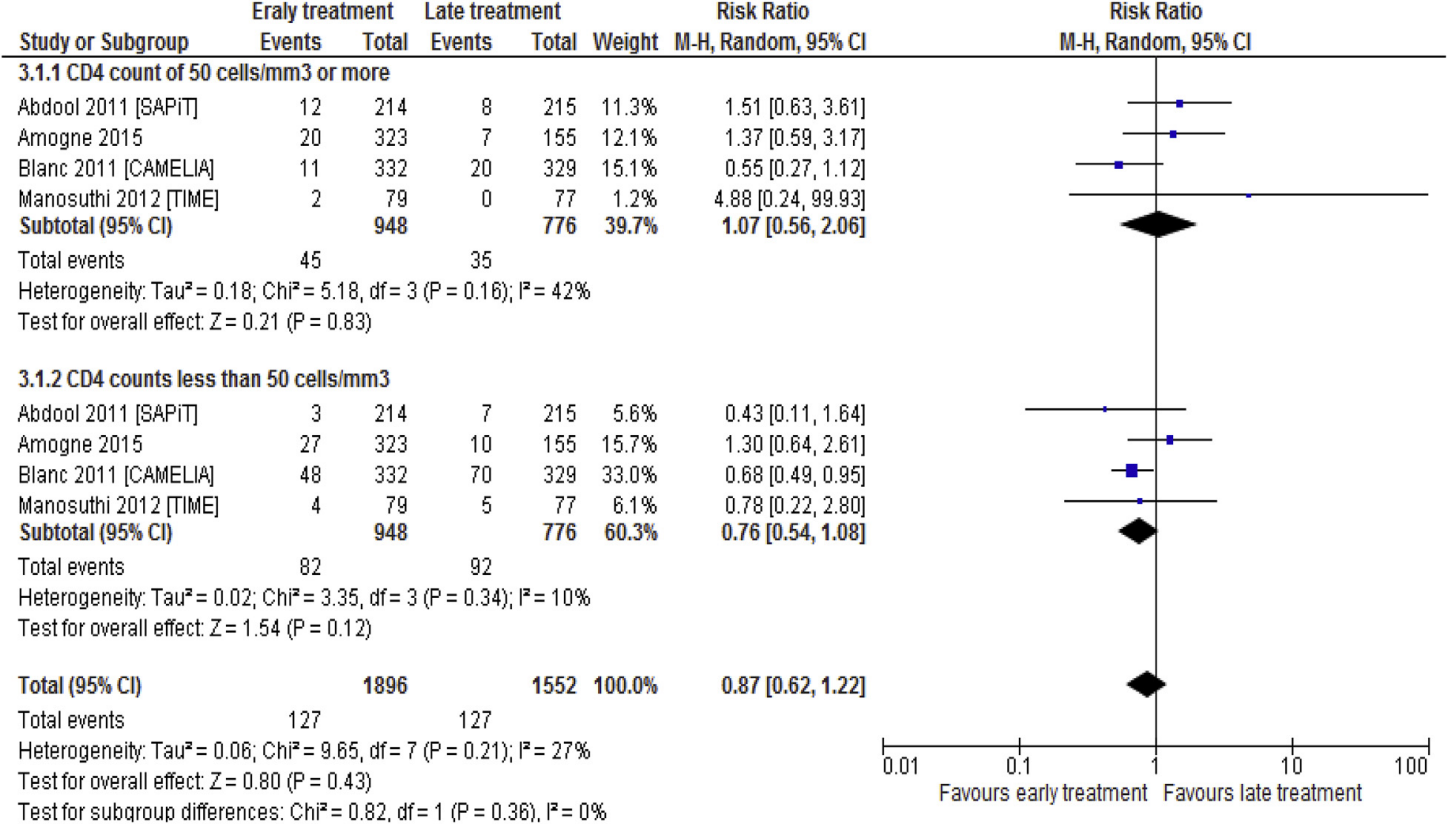
Subgroup analysis based on CD4 counts associated with the effects of time of initiation of antiretroviral therapy in the treatment of adolescent and adult non-pregnant patients with HIV/TB co-infection.

Upon subgroup analysis based on CD4 counts (CD4 < 50 vs. CD4 ≥ 50), there was no statistical significant difference between the two arms with regard to mortality. However, it was clear that mortality was higher in those with a CD4 counts of less than 50 cells/mm3 compared to those with higher CD4 counts in both early and Late ART initiated (Fig. 6).

#### 3.5.2 Grade 3 or 4 adverse events

**Fig. 7:**
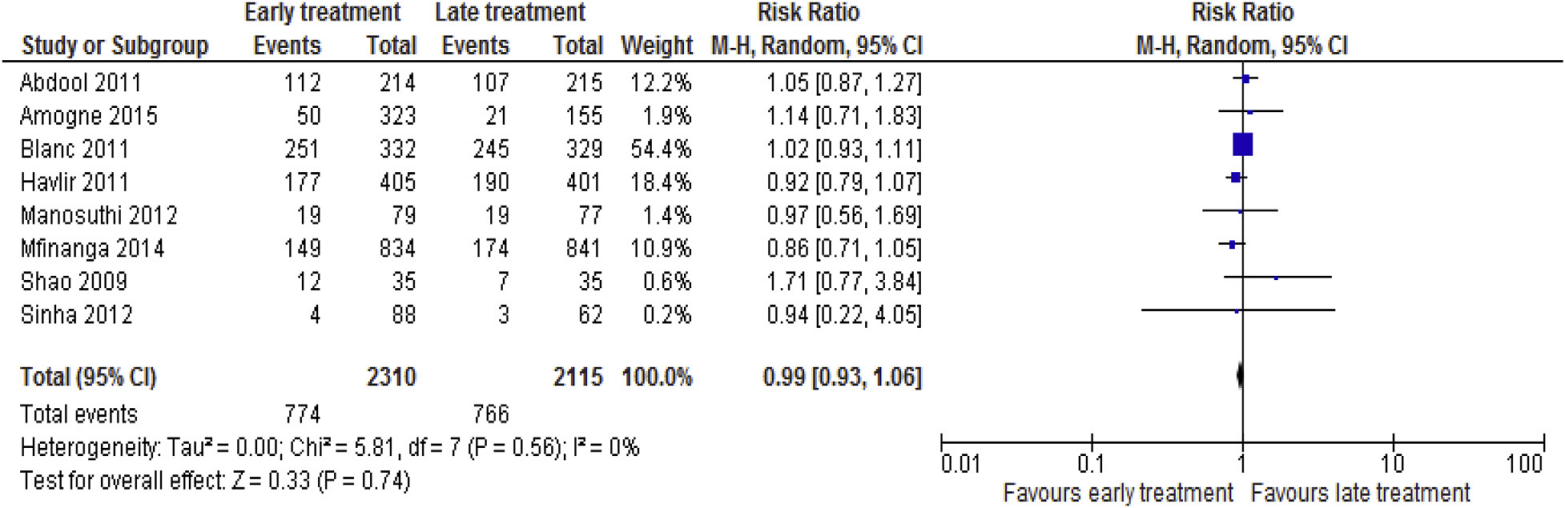
Grade 3 or 4 adverse events associated with the effects of time of initiation of antiretroviral therapy in the treatment of adolescent and adult non-pregnant patients with HIV/TB co-infection.

Data on grade 3 or 4 adverse events for 8 [[18], [19], [20], [21], [22], [23], [24], [25]] trials consisting of 4425 participants were available. No statistical significant difference observed between the two groups with regard to grade 3 or 4 adverse events (RR = 0.99; 95% CI: 0.93–1.06, p = 0.74; I^2^ = 0%, p = 0.56) (Fig. 7).

#### 3.5.3 New AIDS-defining illness

**Fig. 8:**
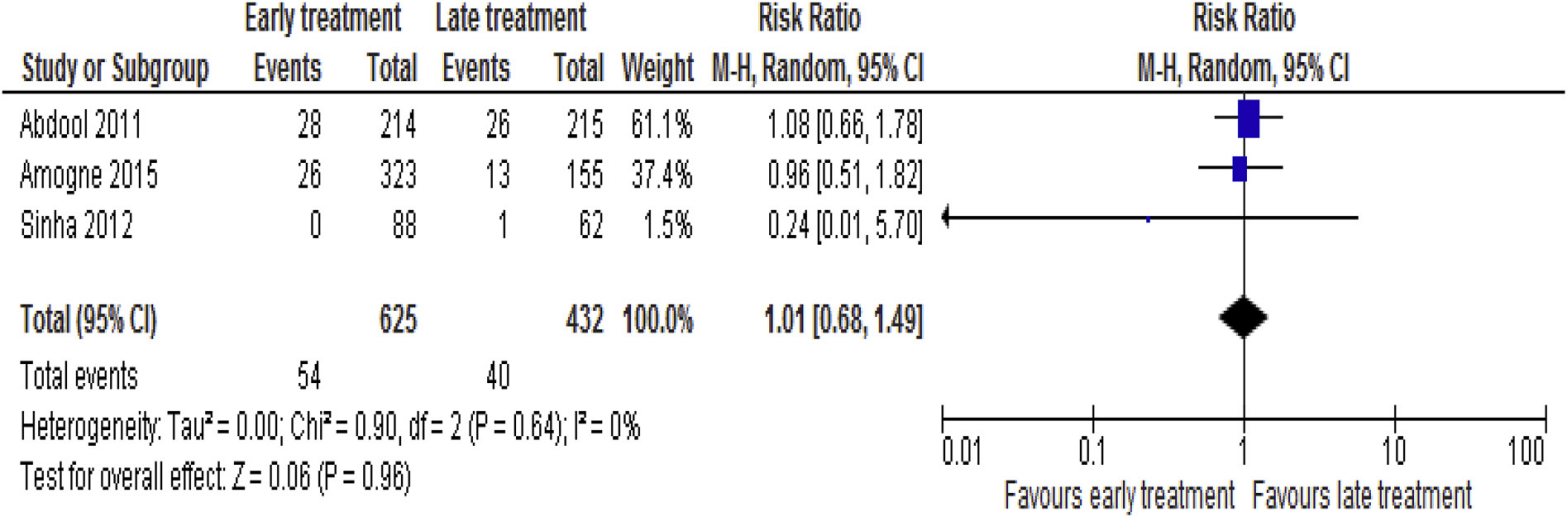
New AIDS-defining illness associated with the effects of time of initiation of antiretroviral therapy in the treatment of adolescent and adult non-pregnant patients with HIV/TB co-infection.

Data for 3 [8, [18], [19], [20]] trails consisting of 1, 057 patients available with respect to the risk of development of new AIDS defining illness. The analysis of the data showed that there was no statistical significant difference between early ART and late ART groups (RR = 1.01; 95% CI: 0.68–1.49, p = 0.96, I^2^ = 0%, p = 0.64) (Fig. 8).

#### 3.5.4 TB treatment failure

**Fig. 9:**
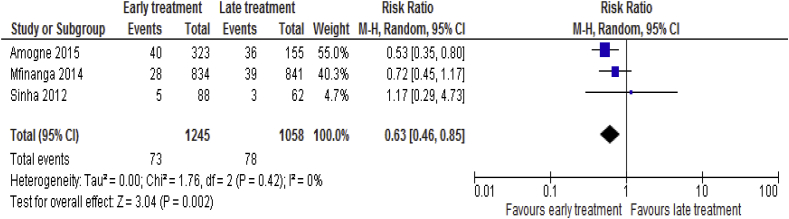
TB treatment failure associated with the effects of time of initiation of antiretroviral therapy in the treatment of adolescent and adult non-pregnant patients with HIV/TB co-infection.

Three [19, 23, 25] trails comprising of 2303 participants include in the analysis for TB treatment failure. The incidence of TB treatment failure in the early ART was 73 (5.9%) versus 78 (7.4%) in the late ART and hence, there were fewer TB treatment failure in the early group compared to the late treatment group (RR = 0.63; 95% CI: 0.46–0.85, p = 0.002; I^2^ = 0%, p = 0.42) (Fig. 9).

#### 3.5.5 TB recurrence

**Fig. 10:**
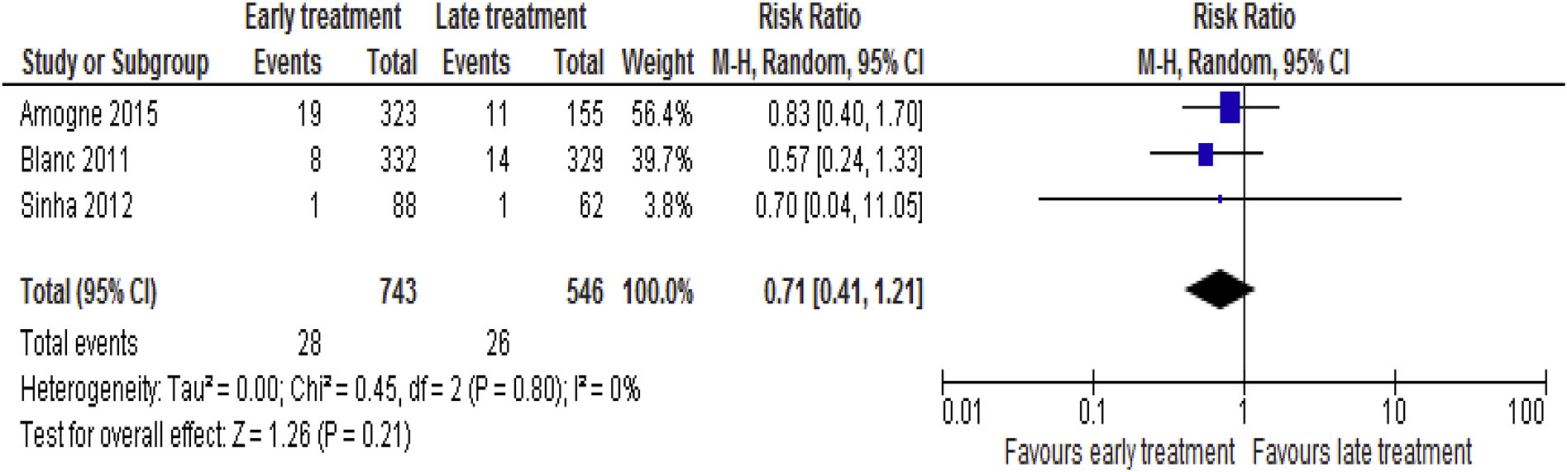
TB recurrence associated with the effects of time of initiation of antiretroviral therapy in the treatment of adolescent and adult non-pregnant patients with HIV/TB co-infection.

Data on TB recurrence was available for 3 [19, 20, 25] trails including 1, 289 patients. The aggregated result of the analysis revealed that there was no statistical significant difference between the two groups (RR = 0.71; 95% CI: 0.41–1. 21, p = 0.21; I^2^ = 0%, p = 0.80) (Fig. 10).

#### 3.5.6 Virological suppression

**Fig. 11:**
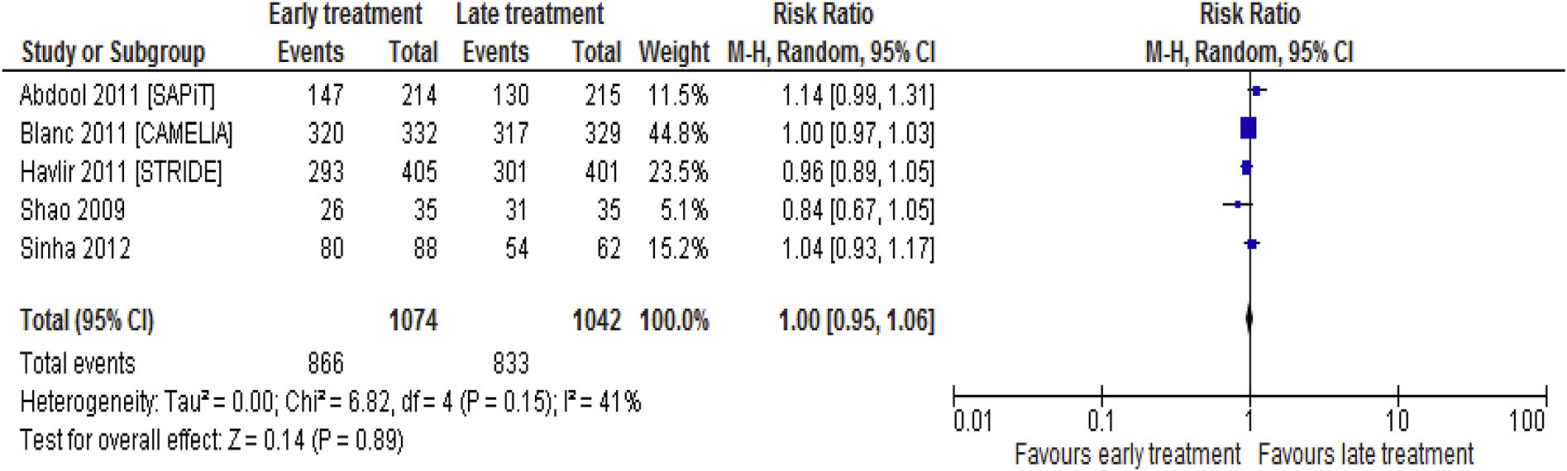
Virological suppression associated with the effects of time of initiation of antiretroviral therapy in the treatment of adolescent and adult non-pregnant patients with HIV/TB co-infection.

Data available on virological suppression (CD4^+^count < 400 copies/mL) for 5 [5, 18, 20, 21, 24, 25]] trials included 2116 participants. No significant difference between the two groups with regard to the degree of viral suppression (RR = 1.00; 95%CI: 0.95 to 1.06, p = 0.89, I^2^ = 41%, p = 0.15) (Fig. 11).

### 3.6 Summary outcomes

The summary of the outcomes in this meta-analysis was depicted in Table 2.

**Table 2:** Summary of statistical outcomes of the meta-analysis of the effects of time of initiation of antiretroviral therapy in the treatment of adolescent and adult non-pregnant patients with HIV/TB co-infection.

Comparison parameters	Endpoint [RR[95% CI]	p- value	Heterogeneity			
			q-value	p-value	I^2^	Tau
Death due to TB- IRIS	6.05[1.06,34.59]	0.04	0.50 (df - 2)	0.78	0%	0.00
Rate of TB-IRIS	1.83[1.24, 2.70]	0.002	23.31(df-6)	0.0007	74%	0.17
Overall mortality	0.81 [0.66, 0.99]	0.04	5.32 (df - 6)	0.50	0%	0.00
Grade 3 or 4 toxicity	0.99[0.93,1.10]	0.74	5.80 (df-7)	0.56	0%	0.00
Viral suppression (<400 copies/mL)	1.00 [0.95, 1.06]	0.89	6.80 (df-4)	0.15	41%	0.00
New AIDS-defining illness	1.01[0.68,1.49]	0.96	0.90 (df-2)	0.64	0%	0.00
TB treatment failure	0.63[0.50,0.90]	0.002	1.76 (df-2)	0.42	0%	0.00
TB recurrence	0.71[0.40,1.20]	0.21	0.45 (df-2)	0.80	0%	0.00

Abbreviations: TB, tuberculosis; IRIS, immune reconstitution syndrome; AIDS, acquired immunodeficiency; Syndrome; df, degree of freedom; RR, risk ratio; CL, confidence interval. Note that results were pooled using random-effect model.

## 4 Discussion

Despite effective therapy for TB and HIV exists, decision to initiate treatment should take into account a number of factors. WHO guidelines [3] on TB–HIV co-treatment prefer deferring ART initiation until TB treatment completion because of concerns of drug interactions between anti-tuberculosis and antiretrovirals [26], occurrence of TB-IRIS [27, 28], side effects (overlapping and/or specific) [29], polypharmacy compromising treatment adherence, and care services challenges [30]. However, delays in ART initiation may result in clinical deterioration due to occurrence of AIDS related illness and death. Therefore, the desired outcome of treating TB–HIV co-infected patients is to plan an optimal balance between the risks and benefits. In the current review, there was a clear evidence of increased incidence of TB-IRIS and death due to TB-IRIS among the group initiated with ART earlier. The absolute risk reduction was 0.78%, which means that for 143 patients treated with ART 8-24 weeks after TB treatment in patients co-infected with HIV and TB; one case of IRIS related mortality would be prevented. In addition, applying the principle of number needed to treat (NNT) showed that 17 patients need to be initiated with ART late in the course of TB treatment to prevent one case of IRIS.

The proposed mechanism for occurrence of TB-IRIS in co-infected patients is related to highly active antiretroviral therapy (HAART)-induced suppression of viral replication and immune recovery, with resultant restoration of TB-specific immune responses [31, 32]. Although, this response is beneficial in most patients, an overly excited host response of some patients may lead to a “paradoxical” TB-IRIS, which is transient clinical deterioration after clinical improvement [33, 34]. Due to lack of gold standard diagnostic test for confirming its diagnosis, TB-IRIS can be under diagnosed or misdiagnosed, resulting in a widely differing incidence in different studies. In addition, in some patients, clinicians may have difficulty distinguishing TB-IRIS from other HIV-related opportunistic diseases, resulting in potentially deleterious consequences for patients [35]. In line with this, our analyses of 8 trials showed that the pooled cumulative incidence of TB-IRIS was 12%.

Early ART resulted in 14.7% TB-IRIS compared with 8.8% in late ART initiation and therefore, had about 2 times more risk of developing TB-IRIS than late ART during TB treatment. This was consistent with report from CAMELIA trials [20, 21]. In this regard, both TB-IRIS and deaths due to TB-IRIS favor late treatment of HIV among TB-HIV co-infected patients. However, the fact that TB-IRIS and deaths due to TB-IRIS were higher in Asian patients emphasizing all ethnicities are not necessarily similar. Another important finding of this study was that the cases of TB-IRIS associated mortality were mainly seen when older antiretroviral drugs such as stavudine or didanosine were used compared with other agents such as zidovudine or tenofovir. Therefore, as these implicated drugs increased TB-IRIS and mortality, they can be avoided or used with caution.

Concurrent use of HAART with anti-tuberculosis in patients with advanced HIV disease is recommended to prevent fatal opportunistic infections [36]. Many observational studies conducted in developing countries have described high mortality rates among HIV-infected TB patients. TB case fatality rates in Africa were 16–35% among HIV-infected patients not receiving ART and up to 4% among HIV-negative individuals [4]. The current recommendation from WHO showed that ART should be initiated for all TB-HIV co-infected patients irrespective of their CD4 counts [37]. However, it should be noted to evaluate effect of early and late ART initiation on overall mortality secondary to progressive immunosuppression. Therefore, our analyses found that there was a mortality benefit of early ART compared with late ART initiation in patients co-infected with TB, a result identical to meta-analysis by Uthman et al. [6] but, disagreed with other trials [21, 38].

In the medical world ‘‘When to initiate ART’’ in patients with HIV-associated TB has been an area of intense debate. This major concern of early initiation of ART was due to overlapping toxicity profiles like hepatotoxicity, cutaneous reactions, renal impairment, neuropathy, and neuropsychiatric adverse effects drug interactions and high pill burden [39]. However, delayed ART initiation may be associated with an increased risk of the AIDS-related illness and death [40, 41]. To evaluate the timing for ART initiation, previous observational studies [42, 43] and RCTs [18, 44] showed that concurrent ART significantly improved survival. Reports of two RCTs provided further evidence to support an early ART initiation (two to four weeks into TB therapy) in the course of anti-tuberculosis treatment, especially in patients with profound immunosuppression [20, 21]. In response to these evidences, 2012 WHO guideline recommended that ART should be initiated as soon as possible within the first eight weeks of anti-tuberculosis treatment. However, recently results from two RCTs in Thailand [22] and India [25] failed to show a significantly survival benefit for patients who initiated ART early posing a potential challenge to WHO recommendations. Our analyses showed that early ART initiation was not associated with an increased risk for grade 3–4 drug-related adverse events. Sensitivity analyses based on different criteria didn't significantly change the pooled results and no statistical heterogeneity was observed among studies.

Despite adequate anti-tuberculosis therapy, many individuals co-infected with TB and HIV had an accelerated course of HIV disease [45]. Since the introduction of HAART, mortality from fatal AIDS defining infections has markedly decreased [46]. However, in the current systematic review and meta-analysis, there was no association between the ART initiation time and the rate of developing new AIDS-defining illness, which was also supported by recent systematic reviews and meta-analysis [12]. Pooled analysis of TB treatment failure in patients treated with anti-tuberculosis medication and ART during TB treatment showed that ART during TB treatment was associated with significantly lower rates of TB failure and relapse [13, 47]. In our current analysis, ART during TB treatment reduced the rates of TB failure which was consistent with previous systematic reviews and meta-analyses [13].

In fact, the current study supports the existing WHO 2016 recommendations to start ART early in patients with CD4 counts of less than 50 cells/mm^3^ for some reasons. First, early ART decreased overall mortality and TB treatment failure. Second, it seems that the increased TB-IRIS and associated deaths were more common with older antiretroviral drugs which are currently almost obsolete even in Sub-Saharan countries. For patients with CD4 greater than 50 cells/mm [3], it is difficult to recommend or refute the early initiation of ART which high lights the importance of clinical judgment by experienced clinician. Additional clinical trials suggested to better define the CD4 threshold at which the mortality benefits of early ART starts to disappear.

### 4.1 Strength and limitation of the study

Strengths of our review include a rigorous search of several databases and other sources to identify eligible randomized, controlled trials and we evaluated individual RCT included for bias introduced by chance through careful review process. The result of this detailed search and analysis calls for the clinical judgment as to the benefits of initiating ART earlier against the risk of TB-IRIS. In addition, the finding of increasing mortality related to TB-IRIS highlights the importance of searching for either preventive or therapeutic regimen.

The study has also some limitations. Caution should be exercised in the interpretation of the results of this analysis due to the nature of the studies included. Only one trial was not subjected to performance bias. The rest 7 trials had unclear risk of blinding participants and personnel. Moreover, the differences in baseline information of the trials included resulted in moderate to high heterogeneity of some of the outcome domains (e.g. incidence of TB-IRS, I [2] = 74%).We are also highly aware of the underestimation of the results of the study due to the exclusion of studies other than English and some vulnerable populations (pregnant women and pediatric patients). Therefore, the conclusion and recommendation of this study is only relevant to the group of population included.

## 5 Conclusion

In summary, the present systematic review and meta-analysis showed evidence that early ART was associated with an increased risk of TB-IRIS and death due to TB-IRIS in patients with TB/HIV co-infection. However, higher rates of TB-IRIS and deaths due to TB-IRIS in Asian patients indicated that all ethnicities are not necessarily similar. The fact that the rate of TB-IRIS and associated mortality higher in patients received either stavudine or didanosine that revealed these drugs can be avoided or used with caution. Moreover, early ART was associated with lower risk of overall mortality and TB treatment failure compared with late ART. Grade 3 or 4 adverse events, achieving lower viral load (<400 copies/mL), TB recurrence rate and development of new AIDS-defining illness were not associated with the time of ART initiation. Hence, the finding of this study calls for clinical judgment as to the benefits of initiating ART earlier against the risk of TB-IRIS and associated mortality. It is also highlights the importance of seeking preventive or therapeutic regimen that can reduce the burden and mortality associated with TB-IRIS.

## Funding

This research did not receive any specific grant from funding agencies in the public, commercial, or not-for-profit sectors.

## Authors' contributions

LCH was involved with study conception and design. LCH, GF, GT and TM were involved in data acquisition. LCH and TM interpreted the data and results of the analyses. LCH and FB drafted the manuscript, which was critically revised by GF and ZM. All authors read and approved the final manuscript.

## Consent for publication

Not applicable. No individual personal details, images or videos are being used in this study.

## Provenance and peer review

Not commissioned, externally peer reviewed.

## Availability of data and materials

Data and all materials of the manuscript are with the author (Legese Chelkeba) and available at any time on request.

## Declaration of competing interest

The authors declare that they have no competing interests.

## Abbreviations

AIDS: acquired immunodeficiency syndrome
ART: antiretroviral therapy
HAART: highly antiretroviral therapy
HIV: human immunodeficiency virus
IRIS: immune reconstitution syndrome
PRISMA: preferred reporting items for systematic reviews and meta-analyses
RCT: randomized clinical trials
RR: risk ratio
TB: mycobacterium tuberculosis
WHO: world health organization

## References

[1] Manosuthi W., Wiboonchutikul S., Sungkanuparph S. (2016). Integrated therapy for HIV and tuberculosis. AIDS Res. Ther..

[2] Gao J., Zheng P., Fu H. (2013). Prevalence of TB/HIV co-infection in countries except China: a systematic review and meta-analysis. PloS One.

[3] World Health Organization (2016). Global Tuberculosis Report 2016. http://www.who.int/tb/publications/global_report/en/.

[4] Mukadi Y.D., Maher D., Harries A. (2001). Tuberculosis case fatality rates in high HIV prevalence populations in sub-Saharan Africa. AIDS.

[5] Török M.E., Yen N.T.B., Chau T.T.H., Mai N.T.H., Phu N.H., Mai P.P., Dung N.T., Chau N.V.V., Bang N.D., Tien N.A. (2011). Timing of initiation of antiretroviral therapy in human immunodeficiency virus (HIV)–associated tuberculous meningitis. Clin. Infect. Dis..

[6] Uthman O.A., Okwundu C., Gbenga K., Volmink J., Dowdy D., Zumla A., Nachega J.B. (2015). Optimal timing of antiretroviral therapy initiation for HIV-infected adults with newly diagnosed pulmonary TuberculosisA systematic review and meta-analysisOptimal timing of ART for HIV-infected adults with TB. Ann. Intern. Med..

[7] Maponga B.A., Chirundu D., Gombe N.T., Tshimanga M., Bangure D., Takundwa L. (2015). Delayed initiation of anti-retroviral therapy in TB/HIV co-infected patients, Sanyati District, Zimbabwe, 2011-2012. Pan Afr. Med. J..

[8] Laureillard D., Marcy O., Madec Y., Chea S., Chan S., Borand L., Fernandez M., Prak N., Kim C., Dim B. (2013). Paradoxical tuberculosis-associated immune reconstitution inflammatory syndrome after early initiation of antiretroviral therapy in a randomized clinical trial. AIDS.

[9] Viskovic K., Begovac J. (2013). Tuberculosis-Associated Immune Reconstruction Inflammatory Syndrome (TB-IRIS) in HIV-Infected Patients: Report of Two Cases and the Literature Overview, Case Reports in Infectious Diseases.

[10] Price P., Murdoch D.M., Agarwal U., Lewin S.R., Elliott J.H., French M.A. (2009). Immune restoration diseases reflect diverse immunopathological mechanisms. Clin. Microbiol. Rev..

[11] Stek C., Schutz C., Blumenthal L., Thienemann F., Buyze J., Nöstlinger C., Ravinetto R., Wouters E., Colebunders R., Maartens G. (2016). Preventing paradoxical tuberculosis-associated immune reconstitution inflammatory syndrome in high-risk patients: protocol of a randomized placebo-controlled trial of prednisone (PredART trial). JMIR Res. Protoc..

[12] Abay S.M., Deribe K., Reda A.A., Baidgilign S., Datiko D., Assefa T., Todd M., Deribew A. (2015). The effect of early initiation of antiretroviral therapy in TB/HIV coinfected patients: A systematic review and meta-analysis. J. Int. Assoc. Provid. AIDS Care (JIAPAC).

[13] Khan F.A., Minion J., Pai M., Royce S., Burman W., Harries A.D., Menzies D. (2010). Treatment of active tuberculosis in HIV-coinfected patients: a systematic review and meta-analysis. Clin. Infect. Dis..

[14] Norman G., Faria R., Paton F., Llewellyn A., Fox D., Palmer S., Clifton I., Paton J., Woolacott N., McKenna C. (2013). The preferred reporting items for systematic reviews and meta-analyses. https://www.ncbi.nlm.nih.gov/books/NBK261239/.

[15] Shea B.J., Reeves B.C., Wells G., Thuku M., Hamel C., Moran J., Moher D., Tugwell P., Welch V., Kristjansson E. (2017). AMSTAR 2: a critical appraisal tool for systematic reviews that include randomised or non-randomised studies of healthcare interventions, or both. BMJ (Clin. Res. Ed.).

[16] Higgins J.P., Altman D.G., Gøtzsche P.C., Jüni P., Moher D., Oxman A.D., Savović J., Schulz K.F., Weeks L., Sterne J.A. (2011). The Cochrane Collaboration's tool for assessing risk of bias in randomised trials. BMJ.

[17] Meintjes G., Lawn S.D., Scano F., Maartens G., French M.A., Worodria W., Elliott J.H., Murdoch D., Wilkinson R.J., Seyler C. (2008). Tuberculosis-associated immune reconstitution inflammatory syndrome: case definitions for use in resource-limited settings. Lancet Infect. Dis..

[18] Abdool Karim S.S., Naidoo K., Grobler A., Padayatchi N., Baxter C., Gray A.L., Gengiah T., Gengiah S., Naidoo A., Jithoo N. (2011). Integration of antiretroviral therapy with tuberculosis treatment. N. Engl. J. Med..

[19] Amogne W., Aderaye G., Habtewold A., Yimer G., Makonnen E., Worku A., Sonnerborg A., Aklillu E., Lindquist L. (2015). Efficacy and safety of antiretroviral therapy initiated one week after tuberculosis therapy in patients with CD4 counts < 200 cells/μL: TB-HAART Study, a randomized clinical trial. PloS One.

[20] Blanc F.-X., Sok T., Laureillard D., Borand L., Rekacewicz C., Nerrienet E., Madec Y., Marcy O., Chan S., Prak N. (2011). Earlier versus later start of antiretroviral therapy in HIV-infected adults with tuberculosis. N. Engl. J. Med..

[21] Havlir D.V., Kendall M.A., Ive P., Kumwenda J., Swindells S., Qasba S.S., Luetkemeyer A.F., Hogg E., Rooney J.F., Wu X. (2011). Timing of antiretroviral therapy for HIV-1 infection and tuberculosis. N. Engl. J. Med..

[22] Manosuthi W., Mankatitham W., Lueangniyomkul A., Thongyen S., Likanonsakul S., Suwanvattana P., Thawornwan U., Suntisuklappon B., Nilkamhang S., Sungkanuparph S. (2012). Time to initiate antiretroviral therapy between 4 weeks and 12 weeks of tuberculosis treatment in HIV-infected patients: results from the TIME study. JAIDS J. Acquir. Immune Defic. Syndr..

[23] Mfinanga S.G., Kirenga B.J., Chanda D.M., Mutayoba B., Mthiyane T., Yimer G., Ezechi O., Connolly C., Kapotwe V., Muwonge C. (2014). Early versus delayed initiation of highly active antiretroviral therapy for HIV-positive adults with newly diagnosed pulmonary tuberculosis (TB-HAART): a prospective, international, randomised, placebo-controlled trial. Lancet Infect. Dis..

[24] Shao H.J., Crump J.A., Ramadhani H.O., Uiso L.O., Ole-Nguyaine S., Moon A.M., Kiwera R.A., Woods C.W., Shao J.F., Bartlett J.A. (2009). Early versus delayed fixed dose combination abacavir/lamivudine/zidovudine in patients with HIV and tuberculosis in Tanzania. AIDS Res. Hum. Retrovir..

[25] Sinha S., Shekhar R.C., Singh G., Shah N., Ahmad H., Kumar N., Sharma S.K., Samantaray J., Ranjan S., Ekka M. (2012). Early versus delayed initiation of antiretroviral therapy for Indian HIV-Infected individuals with tuberculosis on antituberculosis treatment. BMC Infect. Dis..

[26] Piscitelli S.C., Gallicano K.D. (2001). Interactions among drugs for HIV and opportunistic infections. N. Engl. J. Med..

[27] Fishman J.E., Saraf-Lavi E., Narita M., Hollender E.S., Ramsinghani R., Ashkin D. (2000). Pulmonary tuberculosis in AIDS patients: transient chest radiographic worsening after initiation of antiretroviral thrapy. Am. J. Roentgenol..

[28] Chien J.W., Johnson J.L. (1998). Paradoxical reactions in HIV and pulmonary TB. CHEST J..

[29] Girardi E., Palmieri F., Cingolani A., Ammassari A., Petrosillo N., Gillini L., Zinzi D., De Luca A., Antinori A., Ippolito G. (2001). Changing clinical presentation and survival in HIV-associated tuberculosis after highly active antiretroviral therapy. JAIDS J. Acquir. Immune Defic. Syndr..

[30] Karim S.S.A., Karim Q.A., Friedland G., Lalloo U., El Sadr W.M. (2004). Implementing antiretroviral therapy in resource-constrained settings: opportunities and challenges in integrating HIV and tuberculosis care. AIDS.

[31] Mahnke Y.D., Greenwald J.H., DerSimonian R., Roby G., Antonelli L.R., Sher A., Roederer M., Sereti I. (2012). Selective expansion of polyfunctional pathogen-specific CD4+ T cells in HIV-1–infected patients with immune reconstitution inflammatory syndrome. Blood.

[32] Dhasmana D.J., Dheda K., Ravn P., Wilkinson R.J., Meintjes G. (2008). Immune reconstitution inflammatory syndrome in HIV-infected patients receiving antiretroviral therapy. Drugs.

[33] Shelburne S.A., Montes M., Hamill R.J. (2006). Immune reconstitution inflammatory syndrome: more answers, more questions. J. Antimicrob. Chemother..

[34] Lawn S.D., Myer L., Bekker L.-G., Wood R. (2007). Tuberculosis-associated immune reconstitution disease: incidence, risk factors and impact in an antiretroviral treatment service in South Africa. AIDS.

[35] Friedland G. (2009). Tuberculosis immune reconstitution inflammatory syndrome: drug resistance and the critical need for better diagnostics. Clin. Infect. Dis..

[36] Dean G.L., Edwards S.G., Ives N.J., Matthews G., Fox E.F., Navaratne L., Fisher M., Taylor G.P., Miller R., Taylor C.B. (2002). Treatment of tuberculosis in HIV-infected persons in the era of highly active antiretroviral therapy. AIDS.

[37] World Oealth Organization, Initiative S.T. (2010). Treatment of Tuberculosis: Guidelines. http://www.who.int/tb/publications/2010/9789241547833/en/.

[38] Abdool Karim S., Naidoo K., Grobler A., Padayatchi N., Nair G., Bamber S., Pienaar J., Friedland G., El-Sadr W., Karim Q. (2009). Initiating ART during TB Treatment Significantly Increases Survival: Results of a Randomized Controlled Clinical Trial in TB/HIV-co-infected Patients in South Africa, 16th Conference on Retroviruses and Opportunistic Infections.

[39] Tweya H., Ben-Smith A., Kalulu M., Jahn A., Ng’ambi W., Mkandawire E., Gabriel L., Phiri S. (2014). Timing of antiretroviral therapy and regimen for HIV-infected patients with tuberculosis: the effect of revised HIV guidelines in Malawi. BMC Publ. Health.

[40] McIlleron H., Meintjes G., Burman W.J., Maartens G. (2007). Complications of antiretroviral therapy in patients with tuberculosis: drug interactions, toxicity, and immune reconstitution inflammatory syndrome. J. Infect. Dis..

[41] Marks D., Dheda K., Dawson R., Ainslie G., Miller R. (2009). Adverse events to antituberculosis therapy: influence of HIV and antiretroviral drugs. Int. J. STD AIDS.

[42] Velasco M., Castilla V., Sanz J., Gaspar G., Condes E., Barros C., Cervero M., Torres R., Guijarro C. (2009). Effect of simultaneous use of highly active antiretroviral therapy on survival of HIV patients with tuberculosis. JAIDS J. Acquir. Immune Defic. Syndr..

[43] Sungkanuparph S., Manosuthi W., Kiertiburanakul S., Vibhagool A. (2006). Initiation of antiretroviral therapy in advanced AIDS with active tuberculosis: clinical experiences from Thailand. J. Infect..

[44] Abdool Karim S.S., Naidoo K., Grobler A., Padayatchi N., Baxter C., Gray A., Gengiah T., Nair G., Bamber S., Singh A. (2010). Timing of initiation of antiretroviral drugs during tuberculosis therapy. N. Engl. J. Med..

[45] Whalen C., Horsburgh C.R., Hom D., Lahart C., Simberkoff M., Ellner J. (1995). Accelerated course of human immunodeficiency virus infection after tuberculosis. Am. J. Respir. Crit. Care Med..

[46] Mocroft A., Vella S., Benfield T., Chiesi A., Miller V., Gargalianos P., Monforte A.d.A., Yust I., Bruun J., Phillips A. (1998). Changing patterns of mortality across Europe in patients infected with HIV-1. Lancet.

[47] Hermans S.M., Castelnuovo B., Katabira C., Mbidde P., Lange J.M., Hoepelman A.I., Coutinho A., Manabe Y.C. (1999). Integration of HIV and TB services results in improved TB treatment outcomes and earlier, prioritized ART initiation in a large urban HIV clinic in Uganda. J. Acquir. Immune Defic. Syndr..

